# Preventive Effects of Drinking Hydrogen-Rich Water on Gingival Oxidative Stress and Alveolar Bone Resorption in Rats Fed a High-Fat Diet

**DOI:** 10.3390/nu9010064

**Published:** 2017-01-13

**Authors:** Toshiki Yoneda, Takaaki Tomofuji, Muneyoshi Kunitomo, Daisuke Ekuni, Koichiro Irie, Tetsuji Azuma, Tatsuya Machida, Hisataka Miyai, Kouhei Fujimori, Manabu Morita

**Affiliations:** 1 Department of Preventive Dentistry, Okayama University Graduate School of Medicine, Dentistry and Pharmaceutical Sciences, 2-5-1 Shikata-cho, Kita-ku, Okayama 700-8558, Japan; de17057@s.okadai.jp (T.Y.); tomofu@md.okayama-u.ac.jp (T.T.); de19013@s.okayama-u.ac.jp (M.K.); coichiro@md.okayama-u.ac.jp (K.I.); tetsuji@md.okayama-u.ac.jp (T.A.); de17046@s.okadai.jp (T.M.); plzs3rog@okayama-u.ac.jp (H.M.); pyyq1hgd@s.okayama-u.ac.jp (K.F.); mmorita@md.okayama-u.ac.jp (M.M.); 2 Advanced Research Center for Oral and Craniofacial Sciences, Okayama University Dental School, 2-5-1 Shikata-cho, Kita-ku, Okayama 700-8558, Japan

**Keywords:** alveolar bone loss, obesity, oxidative stress, hydrogen-rich water, animal disease model

## Abstract

Obesity induces gingival oxidative stress, which is involved in the progression of alveolar bone resorption. The antioxidant effect of hydrogen-rich water may attenuate gingival oxidative stress and prevent alveolar bone resorption in cases of obesity. We examined whether hydrogen-rich water could suppress gingival oxidative stress and alveolar bone resorption in obese rats fed a high-fat diet. Male Fischer 344 rats (*n *= 18) were divided into three groups of six rats each: a control group (fed a regular diet and drinking distilled water) and two experimental groups (fed a high-fat diet and drinking distilled water or hydrogen-rich water). The level of 8-hydroxydeoxyguanosine was determined to evaluate oxidative stress. The bone mineral density of the alveolar bone was analyzed by micro-computerized tomography. Obese rats, induced by a high-fat diet, showed a higher gingival level of 8-hydroxydeoxyguanosine and a lower level of alveolar bone density compared to the control group. Drinking hydrogen-rich water suppressed body weight gain, lowered gingival level of 8-hydroxydeoxyguanosine, and reduced alveolar bone resorption in rats on a high-fat diet. The results indicate that hydrogen-rich water could suppress gingival oxidative stress and alveolar bone resorption by limiting obesity.

## 1. Introduction

Obesity, defined as abnormal or excessive fat accumulation that increases the risk of chronic disease, has been increasingly linked with periodontal disease. Reports show that individuals who become obese have a higher risk of developing periodontal disease (relative risk (RR) = 1.33, 95% confidence interval (CI) = 1.21–1.47) compared with counterparts of normal weight [1]. Obese individuals have also been shown to have a significantly higher risk of experiencing periodontal disease progression than individuals with normal weight after adjusting for important co-factors (RR = 1.36, 95% CI = 1.04–1.78) [2]. These observations indicate that obesity is a risk for periodontal disease.

Obesity is associated with a systemic increase in reactive oxygen species (ROS) production [3,4]. Although ROS are products of normal cellular metabolism, overproduction of ROS induces oxidative stress by damaging DNA, lipids, and protein [5]. Oxidative stress plays a crucial role in the pathogenesis of a number of diseases, including periodontal disease [6]. In vitro studies have shown that oxidative stress stimulates osteoclast differentiation [7,8]. Animal studies have also suggested that oxidative stress is involved in the progression of alveolar bone resorption [9,10,11,12]. In a recent review, the basis for the relationship between obesity and periodontitis lies at a fundamental intracellular level, which includes oxidative stress [13]. Thus, gingival oxidative stress due to obesity may induce the progression of periodontal disease through increased alveolar bone resorption.

Molecular hydrogen is an antioxidant that can reduce oxidative stress [14], and drinking hydrogen-rich water (HW) can increase the concentration of molecular hydrogen in blood and tissues [15]. In dentistry, animal studies have demonstrated that HW can reduce gingival oxidative stress following aging [16] and periodontal disease [17]. A recent study also revealed that drinking HW activated the gene expression of antioxidant defense, contributing to an acceleration of oral mucosal wound healing in rats [18]. These studies used rats with normal weight. Therefore, the antioxidative effect of HW may offer clinical benefits even in obese rats by limiting obesity-induced oxidative stress. However, it is unclear how HW affects gingival oxidative stress and alveolar bone resorption resulting from obesity.

In the present study, we hypothesized that drinking HW might prevent gingival oxidative stress and alveolar bone resorption in obesity. 8-Hydroxydeoxyguanosine (8-OHdG), which is formed when the guanine in DNA undergoes oxidative damage, is generally accepted as a reliable indicator of oxidative stress [19]. In addition, feeding test subjects a high-fat diet is one of the useful experimental models to investigate periodontal disease progression in obesity [20,21]. The purpose of the present study was to investigate the effects of HW on gingival 8-OHdG levels and alveolar bone resorption in obese rats fed a high-fat diet.

## 2. Materials and Methods

### 2.1. Animals

Male Fischer 344 rats (8 weeks old) were used in this study. The rats were housed in an air-conditioned room (23–25 °C) with a 12 h light–dark cycle. The experiments were performed in accordance with the institutional guidelines of the Animal Research Control Committee of Okayama University (OKA-2014200).

### 2.2. Experimental Design

The rats were randomly divided into three groups of six rats each: For the control group, rats were given pure water (distilled water) and a regular diet (MF, Oriental Yeast Co. Ltd., Osaka, Japan); for the high-fat diet (HFD) group, rats were given pure water and a high-fat diet (F2HFD1, Oriental Yeast Co. Ltd., Osaka, Japan) [22]; for the HFD + HW group, rats were given HW and a high-fat diet. In this study, we did not induce experimental periodontitis in all groups. Pure water or HW was given to the rats every 12 h, morning and night, in a closed glass vessel until they reached 20 weeks old. HW was given immediately after it was prepared. The glass vessel with sipper tube was attached to the cage, and the rats drank water using the sipper tube [16,17]. HW was prepared by electrolysis of water using BLUE OCEAN H2mini (HWP-200WWD, Tech. Co. Ltd., Tokyo, Japan). Water was electrolyzed to afford the following chemical products: 2H_2_O→2H_2_ + O_2_. We measured the hydrogen concentration in HW (1 min and 24 h after electrolysis of water) using a dissolved hydrogen meter (KM2100DH, Kyoei Electronic Laboratory Co. Ltd., Saitama, Japan) three times at each stage. The hydrogen concentration (mean ± standard deviation, µg/L) after 1 min was 301.7 ± 65.1, and that after 24 h was 186.3 ± 55 µg/L. 

After the experimental period, the animals were sacrificed under deep anesthesia with diethyl ether.

### 2.3. Measurements of Serum Parameters

Blood samples were collected from the heart. Serum was separated by centrifugation at 1500× *g* for 15 min and stored at −80 °C until analysis. Serum total cholesterol, very low-density lipoprotein (VLDL) cholesterol, low-density lipoprotein (LDL) cholesterol, high-density lipoprotein (HDL) cholesterol, total triglycerides, VLDL triglycerides, LDL triglycerides, and HDL triglycerides were measured using a gel permeation high performance liquid chromatography system (Skylight Biotech, Akita, Japan) [23]. The level of serum 8-OHdG was also analyzed using an ELISA kit (Japan Institute for the Control of Aging, Shizuoka, Japan) [24].

### 2.4. Measurements of Gingival Level of 8-OHdG

Mitochondrial DNA was isolated from gingival tissue of maxillary molar regions using a DNA extractor kit (Wako Pure Chemical Industries, Osaka, Japan). The level of 8-OHdG in the isolated mitochondrial DNA was analyzed using an ELISA kit (Japan Institute for the Control of Aging, Shizuoka, Japan) [24].

### 2.5. Micro-Computed Tomography (CT) Assessment of Mandible

**Figure 1:**
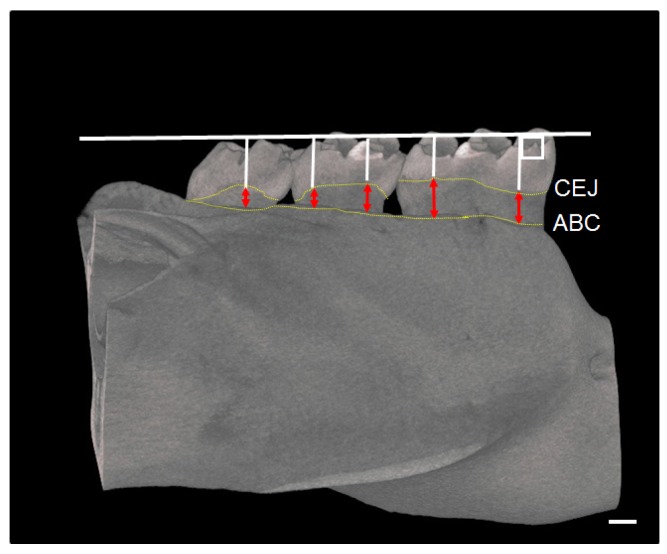
Measurement regions for alveolar bone resorption in rats. The µCT image shows how to measure the distance between CEJ and ABC. The red arrowheads indicate the degree of alveolar bone resorption. CEJ: cemento-enamel junction; ABC: alveolar bone crest. Bar = 500 µm.

Mandibular bones were scanned with a micro-CT device (RmCT, Rigaku, Tokyo, Japan) with the following settings: (1) a slice thickness of 50 μm; (2) a voltage of 90 kV; and (3) an electrical current of 0.1 mA. Three-dimensional images were obtained using a bone analysis system (TRI/3D-BON, Ratoc, Tokyo, Japan). The furcation area of the first molar root was taken for analysis of the percentage of bone volume/total volume (BV/TV%), trabecular number (Tb. N), trabecular thickness (Tb. Th), and trabecular separation (Tb. Sp). The distance between the cemento-enamel junction (CEJ) and the alveolar bone crest (ABC) was measured at 5 points for each mandibular molar (first molar [M1] to third molar [M3]) as alveolar bone resorption [25]. The distances from these 5 points were summed as alveolar bone resorption (Figure 1) [25].

### 2.6. RNA Isolation and PCR Array Analysis

Total RNA of the HFD group and the HFD + HW group was extracted from gingival tissue (100 mg per rat) of the mandibular molar regions using the mirVavaTM PARISTM Kit (Life Technologies, Carlsbad, CA, USA). RNA of the same group was pooled (two rats per one sample). RNA content was measured with a spectrophotometer (Beckman Du 640) (Beckman Coulter, Brea, CA, USA). Total RNA (1 μg RNA from each sample) was used for reverse-transcription with an RT2 First Strand Kit (Qiagen, Hilden, Germany). To profile gene expression, PCR array analysis was performed using an RT2 Profiler PCR Array (rat oxidative stress, PARN-065ZA) (Qiagen) and an RT2 SYBR Green qPCR Master Mix (Qiagen) on an Mx3000P Real-Time QPCR System (Agilent Technologies, Tokyo, Japan). The cycle threshold (*Ct*) values were obtained, and data of the gene expression were analyzed with an online analysis tool (RT2 Profiler PCR array Data Analysis version 3.5, http://pcrdataanalysis.sabiosciences.com/pcr/arrayanalysis.php) (Qiagen). Data were screened for the expression of 84 genes related to oxidative stress. Fold change values up- or down regulation (HFD + HW group/HFD group) were calculated from gene expression (2 − Δ*Ct*).

### 2.7. Statistical Analysis

Data were expressed as means ± standard deviations. A one-way ANOVA followed by Tukey’s method was used for the three-group comparison using a statistical software package (SPSS version 22.0; IBM, Tokyo, Japan). A Student’s *t*-test was used for PCR array analysis.

## 3. Results

### 3.1. Results of Body Weight and Gain of Body Weight

At baseline, there were no significant differences among the three groups with regard to body weight. At 20 weeks old, the HFD group, but not the HFD + HW group, showed greater body weight than did the control group (*p *< 0.05) (Table 1). The body weight gain during the experimental period was higher in the HFD group than that in the control group (*p* < 0.05) and that the HFD + HW group (*p* < 0.05). There was no significant difference in body weight gain between that of the control group and that of the HFD + HW group during the experimental period.

### 3.2. Results of Serum Levels of Cholesterols and 8-OHdG

Serum levels of total cholesterol and VLDL cholesterol in the HFD group and serum levels of VLDL cholesterol in the HFD + HW group were significantly higher than those in the control group (*p* < 0.05) (Table 2). The serum level of total triglycerides in the HFD + HW group was significantly lower than that in the control group (*p* < 0.05). Serum levels of VLDL triglycerides were significantly lower in the HFD group in the HFD + HW group than those in the control group (*p* < 0.05). On the other hand, no significant differences in serum cholesterol and triglyceride between those in the HFD and the HFD + HW groups were found. In addition, serum levels of 8-OHdG in the HFD group were significantly higher than those in the control group (*p* < 0.05), and those in the HFD + HW group was significantly lower than those in the HFD group (*p* < 0.05).

### 3.3. Results of Gingival Level of 8-OHdG

**Figure 2:**
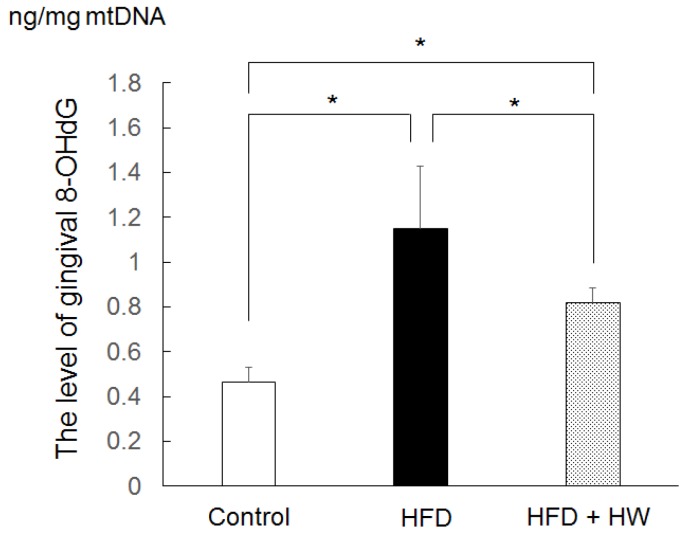
The level of gingival 8-OHdG in rats. Values are presented as the mean ± standard deviation of six rats. * *p* < 0.05, using Tukey’s methods.

Gingival levels of 8-OHdG in the HFD group and the HFD + HW group were higher than those in the control group (*p* < 0.05) (Figure 2). The gingival level of 8-OHdG in the HFD + HW group was also lower than that in the HFD group (*p* < 0.05).

### 3.4. Results of Micro-CT Analyses of Mandibular Bone

**Figure 3:**
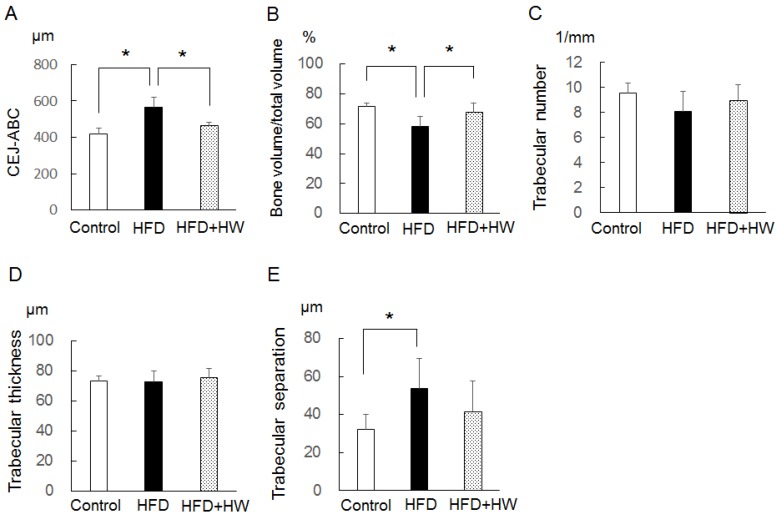
Bone morphogenetic analyses of rat mandible at 20 weeks old. (**A**) Distance between CEJ-ABC; (**B**) percentage of bone volume/total volume; (**C**) trabecular number; (**D**) trabecular thickness; (**E**) trabecular separation. Values are presented as the mean ± standard deviation of six rats. * *p* < 0.05, using Tukey’s methods.

The HFD group, compared with the control group, showed a greater distance between the CEJ-ABC (*p* < 0.05), a lower BT/TV (*p* < 0.05), and a higher Tb. Sp (*p* < 0.05) (Figure 3). The HFD + HW group, compared with the HFD group, also showed a lower distance between the CEJ-ABC (*p* < 0.05) and a greater BT/TV (*p* < 0.05), and these values did not differ from the control group. As for Tb. N and Tb. Th, there were no significant differences among the three groups.

### 3.5. Results of Changes in Oxidative Stress-Related Gene Expression

In the PCR array analysis, four genes involved in oxidative stress showed significant differential gene expression between the HFD and HFD + HW groups, with the values of fold upregulation (>2) or downregulation (<−2) (Table 3). Of these, three genes (isocitrate dehydrogenase 1 (Idh1), superoxide dismutase (Sod) 2, and Sod3) were upregulated and one gene (Fanconi anemia group C (Fancc)) was downregulated in the HFD + HW group. This section may be divided by subheadings. It should provide a concise and precise description of the experimental results, their interpretation, and the experimental conclusions that can be drawn.

## 4. Discussion

In the present study, the rats on a high-fat diet, compared with the control rats, showed increased body weight gain and higher levels of serum and gingival 8-OHdG at 20 weeks. 8-OHdG is an indicator of oxidative stress [18]. These findings indicate that, in the obese rats, a high-fat diet induced a systemic increase in oxidative stress. On the other hand, drinking HW suppressed the effects of a high-fat diet on body weight gain, and showed lower levels of serum and gingival 8-OHdG compared with the obese rats. It is feasible that HW suppresses obesity, which in turn might inhibit a systemic increase in oxidative stress, including gingival oxidative stress.

Some studies have investigated the anti-oxidative effects of HW in obesity. For instance, one animal study has revealed that drinking HW reduced obesity and reduced hepatic oxidative stress in a mice model [26]. A clinical study also showed that consumption of HW decreased the level of urinary lipid peroxidation significantly from baseline to Week 8 in obese subjects [27]. These are consistent with our findings, which exhibited that drinking HW prevented obesity and gingival oxidative stress.

In our findings, the obese rats on a high-fat diet showed a greater distance between the CEJ-ABC, a lower BT/TV, and a higher Tb. Sp than the control rats, suggesting that the progression of alveolar bone resorption follows obesity. The results also revealed that the rats fed a high-fat diet and HW, compared with the HFD rats showed a lower distance between the CEJ-ABC and a greater BT/TV. Gingival oxidative stress is involved in the progression of alveolar bone resorption [11,12]. Suppression of obesity by drinking HW may attenuate alveolar bone resorption by limiting gingival oxidative stress.

This study exhibited that drinking HW upregulated the gene expression of Sod2 and Sod3 in gingival tissues. Sod2 and Sod3 are antioxidant enzymes that catalyze the conversion of superoxide radicals into hydrogen peroxide and oxygen [28,29]. This suggests that the increased antioxidative properties of gingival tissues by HW might also protect periodontal tissues from a systemic increase in oxidative stress.

The present results also showed that HW downregulated the gene expression of Fancc. Since Fancc is considered an oxidative stress responsive gene [30], and it is suggested that the downregulation of Fancc reflected decreased gingival oxidative stress. 

It is reported that drinking HW can improve cholesterol metabolism [27], which may have a direct influence on bone resorption [31]. However, in our findings, there were no significant differences in serum cholesterol and triglyceride between the rats with and without HW intake. These suggest that gingival oxidative stress and alveolar bone resorption were not related to serum cholesterol and triglyceride in our animal model.

In this study, the rats on a high-fat diet tended to exhibit lower levels of total and VLDL triglycerides in serum compared to the control rats. These findings are similar to those of the previous study in birds on a high-fat diet [32]. The reduced serum triglyceride concentrations may reflect a possible metabolic overcompensation in response to the added dietary fat [33].

A clinical study showed that obesity may influence periodontal tissue destruction and disease severity by increasing the level of oxidative stress in the presence of periodontal disease [34]. Another study also suggested that periodontal oxidative stress generated by obesity seems to be associated with periodontal disease [35]. Such evidence supports the concept that obesity may lead to the progression of periodontal disease by increasing oxidative stress. On the other hand, the present report showed that drinking HW suppressed obesity, gingival oxidative stress, and alveolar bone resorption in rat models. Our previous study also found that exercise training prevented obesity and gingival oxidative stress in rats on a high-fat diet [22]. Anti-obesity therapy to reduce oxidative stress may be useful in preventing periodontal disease related to obesity.

In our previous study, broccoli supplementation inhibited the effects of a high-cholesterol diet on osteoclast differentiation in the periodontal tissue [36]. It is also reported that supplementation of coenzyme 10 counteracted the negative effects of n-6 polyunsaturated fatty acid on age-related alveolar bone loss [37]. Furthermore, the present study demonstrated that drinking HW attenuates alveolar bone resorption related to obesity. Action of adequate nutrient consumption would be important to maintain and/or improve periodontal health. 

When taking medicine, it is necessary to be careful about side effects. However, HW has no known side effects in previous animal and human studies [16,17,18,27]. Therefore, HW may constitute useful preventive treatment against periodontal disease related to obesity without side effects.

Our study has some limitations. For instance, no data were collected with regard to the circulating inflammatory molecules, such as tumor necrosis factor-α. Obesity could indirectly result in an increase in the circulation of inflammatory molecules that could augment the inflammation induced by bacterial pathogens. In addition, while we showed preventive effects of HW on obesity and gingival oxidative stress, we could not fully elucidate the relationship between these pathological mechanisms and the effects of HW. Further studies are needed to clarify this point.

## 5. Conclusions

In conclusion, within the limits of the study, drinking HW can inhibit gingival oxidative stress induced by obesity, and thus prevent alveolar bone resorption in rat models. However, clinical trials will be necessary to clarify whether drinking HW can prevent obesity-related complications, including periodontal disease.

## Figures and Tables

**Table 1:** Changes in body weight during the experimental period.

	Control	HFD	HFD + HW
body weight (baseline) (g)	269 ± 12	278 ± 14	276 ± 9
body weight (20 weeks old) (g)	338 ± 14	360 ± 16 *	342 ± 12
body weight gain (20 weeks old—base line) (g)	69 ± 8	82 ± 10 *	66 ± 7 ^†^

Values are presented as the mean ± standard deviation of six rats. * *p* < 0.05 compared with control group, ^†^ *p* < 0.05 compared with the HFD group. The *p*-value was calculated by Tukey’s methods.

**Table 2:** Serum parameters.

	Control	HFD	HFD + HW
total cholesterol (mg/dL)	43.2 ± 24.8	101.1 ± 53.7 *	94.3 ± 18.0
VLDL cholesterol (mg/dL)	5.2 ± 3.3	34.6 ± 20.3 *	34.1 ± 6.4 *
LDL cholesterol (mg/dL)	14.9 ± 9.6	15.6 ± 7.7	16.8 ± 4.2
HDL cholesterol (mg/dL)	21.7 ± 11.5	27.3 ± 11.9	27.9 ± 5.2
total triglycerides (mg/dL)	65.3 ± 46.8	27.6 ± 14.3	19.1 ± 5.3 *
VLDL triglycerides (mg/dL)	43.3 ± 29.5	14.2 ± 8.0 *	10.1 ± 3.4 *
LDL triglycerides (mg/dL)	4.88 ± 3.4	2.86 ± 1.3	1.98 ± 0.4
HDL triglycerides (mg/dL)	3.6 ± 2.1	3.0 ± 1.1	2.6 ± 0.5
8-OHdG (ng/mL)	0.12 ± 0.03	0.17 ± 0.05 *	0.12 ± 0.03 ^†^

Values are presented as the mean ± standard deviation of six rats. * *p* < 0.05 compared with control group, ^†^ *p* < 0.05 compared with the HFD group. The *p*-value was calculated by Tukey’s methods.

**Table 3:** Differentially expressed genes involved in oxidative stress of gingival tissue between the HFD group and the HFD + HW group.

Gene Symbol	Description	Fold Up- or Down Regulation	*p*-Value
		(HFD+HW Group/HFD Group)	
Idh1	isocitrate dehydrogenase (NADP(+)) 1, cytosolic	4.04	0.014
Sod2	superoxide dismutase 2	2.25	0.032
Sod3	superoxide dismutase 3	3.35	0.034
Fancc	Fanconi anemia, complementation group C	−2.88	0.046

The *p*-value was calculated by Student’s *t*-test.
